# Evaluation of adherence to the Mediterranean diet with sustainable nutrition knowledge and environmentally responsible food choices

**DOI:** 10.3389/fnut.2023.1158155

**Published:** 2023-04-12

**Authors:** Emine Yassıbaş, Hatice Bölükbaşı

**Affiliations:** Department of Nutrition and Dietetics, Faculty of Health Sciences, Gazi University, Ankara, Türkiye

**Keywords:** sustainable nutrition, Mediterranean diet, environmentally responsible food, nutrition knowledge, healthy nutrition

## Abstract

**Background:**

Dietary patterns and their possible effects on health and the environment are becoming increasingly important. It is thought that nutritionally balanced diets can also be compatible with environmental targets and, therefore, the Mediterranean diet (MD), which is regarded as a sustainable diet model, comes to the fore. This study was carried out to evaluate adherence to the MD with sustainable nutrition knowledge and environmentally responsible food choices and to determine the factors affecting adherence.

**Methods:**

A questionnaire prepared by the researchers was sent to individuals online and 1732 adults living in Turkey participated in this cross-sectional study. Adherence to the MD was evaluated with the Mediterranean Diet Adherence Screener (MEDAS). In addition, questions were asked about nutritional knowledge and environmentally responsible food choices to evaluate the sustainable nutritional behaviors of individuals.

**Results:**

Half of the participants (51.1% of men / 53% of women) adhere to the MD at a moderate level. Even the individuals with the highest adherence to the MD had low compliance with the recommendations for fruit (43.4%) and fish (37.3%) consumption. A one-unit increase in age, sustainable nutrition knowledge score, and environmentally responsible food choices score increases the MD adherence score by 0.08, 0.125, and 0.148 points, respectively (*p* < 0.005). Individuals with high adherence to the MD avoid consuming genetically modified organism food more (*p* < 0.001), prefer to consume environmentally labeled foods (*p* < 0.001), and buy food more from local businesses (*p* < 0.001), while they prefer to buy imported food less (*p* = 0.034).

**Conclusion:**

The results of this study showed that some strategies should be developed to increase the adaptation of individuals to the MD and sustainable nutritional behaviors. Nutritionally adequate, sustainable, and eco-friendly nutritional behaviors should be encouraged to increase the possible health benefits of nutrition and minimize environmental effects. To promote sustainable nutrition, firstly it is important to determine the knowledge level of individuals concerning sustainable nutrition and, for this purpose, it is thought that an international valid sustainable nutrition knowledge assessment tool is needed.

## 1. Introduction

As a result of the increase in the world’s population, natural resources are faced with the danger of depletion, and thus issues such as social inequality, environmental degradation, and climate change have begun to be addressed within the framework of ‘sustainability’ (1). In this context, it is aimed to meet the needs of current generations without compromising the ability of the next generations to meet their own needs (2).

Nutrition plays a key role in promoting healthy and sustainable diets and ensuring food security globally in achieving the “zero hunger” goal, which is among the 17 sustainable development goals (3). Increasing global population, developing technology, urbanization, and changes in consumption patterns cause individuals’ nutritional habits to change. Traditional diets are being replaced by diets in which more refined sugars, fats, and meats are consumed. These dietary changes greatly increase the incidence of type 2 diabetes, coronary heart disease, and other chronic diseases that reduce global life expectancy, while also increasing greenhouse gas emissions and use of land, livestock, water, and agrochemical (4, 5). Recent studies focus on nutritional habits and dietary patterns and their effects on health, the environment, and food system (5–7). With the concept of a sustainable diet, initiatives are planned to eradicate poverty, food and nutrition insecurity, and poor health outcomes (8).

A sustainable diet contributes to food and nutrition security and healthy life for current and future generations; has a low environmental impact; is culturally acceptable, accessible, affordable, nutritionally sufficient, safe, and healthy; makes the best use of natural and human resources; and respects ecosystems (9). There is evidence that nutritionally balanced diets may also be compatible with environmental goals (10). The Mediterranean diet (MD) has four main sustainability dimensions: health and nutrition benefits, low environmental impact and rich biodiversity, cultural heritage with high sociocultural food values, and positive local economic returns (11, 12). The MD is a dietary pattern characterized by high consumption of olive oil, vegetables, fruits, legumes, whole grains, and nuts; moderate whole milk and dairy products, wine, eggs, chicken, fish, and seafood (depending on the proximity of the population to the seashore); and low consumption of red meat, saturated fat and sweets, and its main feature is nutritional diversity (13).

The MD reduces environmental impact by focusing on increased consumption of plant-based foods compared to animal-derived and processed foods. It has a positive effect on environmental sustainability as it has a lower carbon and water footprint, lower greenhouse gas emissions, and less land and energy use compared to existing Western-type dietary patterns (5, 6). It provides a reduction in water and fuel consumption by reducing the consumption of processed food, and it also supports the consumption of local products, especially by including fish and seafood in the diet (5). It is predicted that transition to the MD will reduce environmental impact (−72%), land use (−58%), energy use (−52%), and water consumption (−33%) (14).

The MD is a healthy eating pattern involving interactions with cultures, people, and the environment, as well as sustainability. Educating the younger generations about the benefits of healthy and sustainable diets and raising consumer awareness of local and sustainable foods will be the first and easiest steps towards achieving global goals (15). It is extremely important to consume healthy and eco-friendly foods, to prevent non-communicable diseases (which are one of the main causes of death in the world) for the protection of human health, and to prevent excessive use of natural resources, climate change, and pollution in order to protect the health of the planet (16).

In the literature, studies on the MD as a sustainable dietary pattern and its possible effects on the environment are increasing (6, 15). However, to the best of our knowledge, there has been no study examining the relationship between individuals’ knowledge of sustainable nutrition and their adherence to the MD. At this point, it is important to investigate the role of individuals’ adherence level to the MD and their level of knowledge about sustainable nutrition in this adherence. It is thought that our study would be important in terms of filling this gap in the literature. Furthermore, practices such as eco-labeling are considered an important indicator of the increase in individuals’ awareness of environmentally responsible foods and their interest in these products (17). Therefore, it is regarded as essential to evaluate the preferences of individuals for traditional/local foods, products with environmental labels, and especially ultra-processed foods in food preferences within the framework of sustainable nutrition.

Accordingly, in the present study it was aimed to evaluate adherence to the MD among adults as a sustainable dietary pattern and to determine the factors affecting adherence to this diet, especially sustainable nutrition knowledge and environmentally responsible food choices.

## 2. Materials and methods

### 2.1. Participants

Individuals selected by snowball sampling living in different cities in Turkey participated in this cross-sectional study. A pilot study was conducted with 15 individuals to determine the sample size. With the data obtained from the pilot study, the sample size was determined as at least 100 individuals using the program Power Analysis and Sample Size (PASS), provided that alpha (*α*) = 0.05, power (1–*β*) = 0.95, and the correlation coefficient was 0.35.

### 2.2. Ethical considerations

In order to carry out the research, approval was obtained from Gazi University Ethics Committee (Research Code No: 2021–04). Volunteers who agreed to participate in the study were informed about the study and their consent was obtained. This study was conducted by the principles of the Declaration of Helsinki.

### 2.3. Data collection

The data were collected through an online questionnaire sent to the participants. In the first part of the questionnaire, there were questions about sociodemographic characteristics (age, sex, educational status, etc.). The Mediterranean Diet Adherence Screener (MEDAS) was used to evaluate adherence to the MD, which is accepted as a sustainable nutritional model. In addition, questions were asked about nutritional knowledge and environmentally responsible food choices to evaluate the sustainable nutritional behaviors of individuals. The body weight and height of the individuals were recorded based on the declaration. Body mass index (BMI) was calculated and evaluated according to the classification of the World Health Organization.

### 2.4. Data collection tools

#### 2.4.1. Mediterranean diet adherence screener

Mediterranean Diet Adherence Screener, which was developed by Martínez-González et al. (18) in 2012, was adapted into Turkish by Pehlivanoğlu et al. (19). It is a valid and reliable tool for assessing adherence to the MD. The habits of consuming foods that are characteristic of the MD are established with two questions on the scale and food consumption frequency with 12 questions. Each question is scored as 0 or 1 point. The total score ranges from 0 to 14, with 0 indicating the lowest adherence to the MD and 14 the highest adherence. An MD adherence score of ≤ 5 indicates low adherence, 6–9 moderate adherence, and ≥ 9 high adherence (19).

#### 2.4.2. Sustainable nutrition knowledge

To determine sustainable nutrition knowledge, 15 items (in Supplementary material) prepared by Gülsöz (20) for her master’s thesis, using international literature, were used. The calculation was made by giving 1 point to the correct answer for each item. The highest score that can be obtained in total is 15, and a higher score indicates greater sustainable nutrition knowledge (20).

#### 2.4.3. Environmentally responsible food choices

To evaluate the environmentally responsible food choices of individuals, the 7-item “Environmentally Responsible Food Choices” instrument developed by Başar was used (17). The items are as follows: “I can pay more for organically grown food,” “I avoid consuming food with genetically modified organisms (GMOs),” “I prefer to consume eco-label food,” and “I am careful not to consume too much meat,” “I prefer to buy dairy products from local producers,” “I avoid consuming imported food such as a variety of exotic fruits,” and “I avoid consuming canned “ready-made” food.” Item responses are 5-point Likert type and each item is scored between 1 and 5. The total score is at most 35 and a higher score indicates more environmentally responsible food choices.

#### 2.4.4. Statistical analysis

Statistical analyses of the data obtained were conducted using IBM SPSS Statistics 22 (IBM SPSS, Turkey). In the descriptive analyses, categorical data were used as numbers and percentages, mean, and standard deviation values were used according to the normality of the numerical data. Compliance with the normal distribution was examined by Kolmogorov–Smirnov/Shapiro–Wilk tests and histogram plot. The Chi-square test was used to compare the categorical data. For the numerical data, the t-test was used to compare two groups. A multiple linear regression model was used to identify independent predictors of the MD. The differences in compliance rates for each food and food group according to the MEDAS items were calculated and the difference between the current status (percentage of participants currently adhering to each dietary recommendation) and the ideal situation (100% compliance) is shown in radar charts. The statistical significance level was set at 0.05.

## 3. Results

The study was conducted with 1732 adults and their general characteristics are shown in Table 1. The mean age of the participants was 27.7 ± 10.00 years, and the majority (72.1%) were between 18 and 29 years old. Most of the participants (78.1%) were university students/graduates. The mean BMI was 22.8 ± 4.21 kg/m^2^ in women and 25.3 ± 4.07 kg/m^2^ in men and 59.2% of all participants had a normal BMI (Table 1).

**Table 1:** Sociodemographic characteristics of the participants.

Sociodemographic characteristics	Men (*n* = 615)		Women (*n* = 1.117)		Total (*n* = 1732)	
	*n*	%	*n*	%	*n*	%
*Age (years)*						
18–29	387	62.9	862	77.2	1.249	72.1
30–39	76	12.4	153	13.7	229	13.2
40–49	78	12.7	63	5.6	141	8.1
≥50	74	12.0	39	3.5	113	6.5
*Age (years)* (mean ± SD)	30.7 ± 12.05		26.1 ± 8.24		27.7 ± 10.00	
*Educational level*						
Primary school	7	1.1	23	2.1	30	1.7
Secondary school	11	1.8	24	2.1	35	2.0
High school	97	15.8	110	9.8	207	12.0
University	451	73.3	901	80.7	1.352	78.1
Postgraduate	49	8.0	59	5.3	108	6.2
Length of education (years; mean ± SD)	15.3 ± 2.20		15.3 ± 2.28		15.3 ± 2.25	
*Body Mass Index (BMI) (kg/m^2^)*						
<18,5 (underweight)	20	3.3	127	11.4	147	8.5
18,5–24,9 (normal)	289	47.0	736	65.9	1.025	59.2
25,0–29,9 (overweight)	224	36.4	183	16.4	407	23.5
>30 (obese)	82	13.3	71	6.4	153	8.8
*Body Mass Index (BMI) (kg/m^2^)* (mean ± SD)	25.3 ± 4.07		22.8 ± 4.21		23.7 ± 4.33	

The participants’ MEDAS scores and levels, sustainable nutrition knowledge scores, and environmentally responsible food choices scores are given in Table 2. It was determined that 51.1% of men and 53% of women showed moderate adherence to the MD. While there was no significant difference between the MEDAS scores according to sex (*M* = 6.76 ± 2.08; *W* = 7.01 ± 2.06 *p* > 0.05), sustainable nutrition knowledge (*M* = 8.43 ± 2.72; *W* = 9.25 ± 2.51, *p* < 0.05) and environmental responsible food choices scores (*M* = 22.36 ± 5.40; *W* = 22.67 ± 4.81, *p* < 0.05) were higher in the women than in the men. According to the level of adherence to the MD, the answers given by the participants regarding sustainable nutrition knowledge and environmentally responsible food choices are given in Supplementary Table 1 and Table 2.

**Table 2:** Adherence to the Mediterranean diet, sustainable nutrition knowledge and environmentally responsible food preference scores of the participants.

	Men (*n* = 615)		Women (*n* = 1.117)		Total (*n* = 1732)	
	*n*	%	*n*	%	*n*	%
*Level of adherence to the Mediterranean diet*						
Low adherence	170	27.6	257	23.0	427	24.7
Moderate adherence	314	51.1	592	53.0	906	52.3
High adherence	131	21.3	268	24.0	399	23.0
	*p* = 0.083^*^					
Adherence to the Mediterranean diet scores (mean ± SD)	6.76 ± 2.08		7.01 ± 2.06		6.92 ± 2.07	
	*p* = 0.233^**^					
Sustainable nutrition knowledge scores (mean ± SD)	8.43 ± 2.72		9.25 ± 2.51		8.96 ± 2.62	
	***p* = 0.015** ^**^					
Environmentally responsible food preference scores (mean ± SD)	22.36 ± 5.40		22.67 ± 4.81		22.56 ± 5.03	
	***p* = 0.00** ^**^					

* Chi-square test.

** Student *t* test.

The bold values indicate statistically significant values (*p* < 0.05).

**Figure 1:**
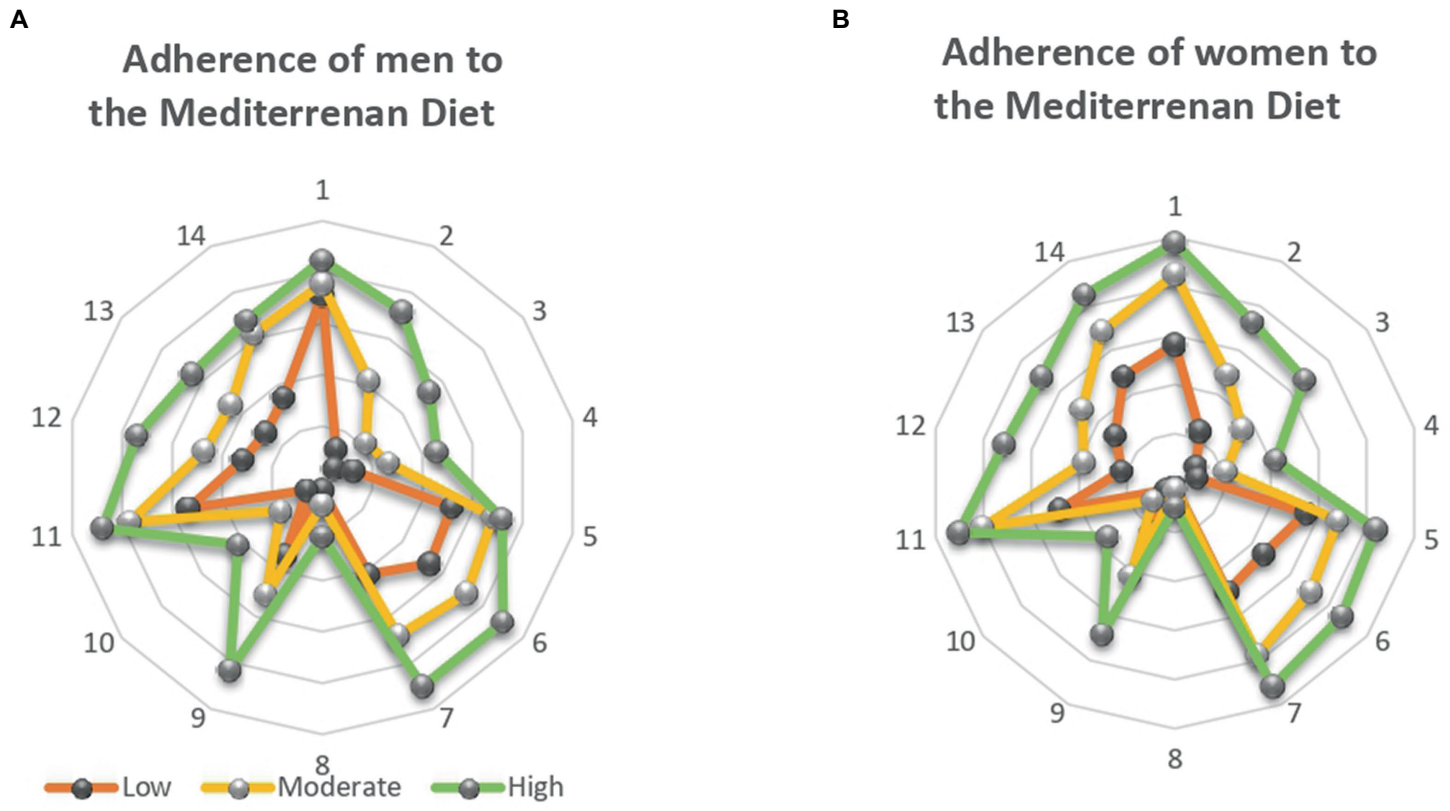
Compliance to Mediterranean diet adherence screener (MEDAS) items according to sex **(A)** men, **(B)** women, and level of adherence to the Mediterranean diet.

The compliance of the men and women with MEDAS items according to their adherence to the MD is shown in Figure 1. The use of olive oil as the main oil type was high only in the group with high adherence to the MD among the women. However, olive oil use was high in all men, regardless of MD adherence. Even the men and women who showed the highest adherence to the MD had low compliance (below 50%) with the recommendations for fruit (43.4%) and fish consumption (37.3%; Figure 1; Supplementary Table 3). Moreover, 58% of the men and 64.9% of the women who adhered closely to the MD did not comply with the recommendation regarding fish consumption. Individuals were in greater compliance with the recommendation of low consumption of butter/margarine, sweet or carbonated beverages, and bakery products (Supplementary Table 3). In addition, compliance with the recommendations for red wine consumption was low in both sexes (Figure 1).

Regression models were created to identify potential factors affecting individuals’ adherence to the MD (Supplementary Table 4). In the first stage, sex, age, and BMI and, afterward, length of education, sustainable nutrition knowledge score, and environmentally responsible food choices score were added to the models. In the last stage, model 5 was created with the significant parameters. According to model 5, a one-unit increase in age, sustainable nutrition knowledge score, and environmentally responsible food choices score increases the MD adherence score by 0.08, 0.125, and 0.148 points, respectively (*p* < 0.005). In the multilinear model created, sex, age, sustainable nutrition knowledge score, and environmentally responsible food choices score with a 5% change in MD adherence score are explained (Table 3).

**Table 3:** Regression models for potential factors affecting adherence to the Mediterranean diet.

	Model 1			Model 2			Model 3			Model 4			Model 5		
	*β*	*se*	*p*	*β*	*se*	*p*	*β*	*se*	*p*	*β*	*se*	*p*	*β*	*se*	*p*
Sex	0.077	0.108	0.002	0.081	0.109	0.001	0.063	0.109	0.012	0.053	0.108	0.033	0.053	0.105	0.030
Age	0.123	0.006	<0.001	0.136	0.006	<0.001	0.130	0.006	<0.001	0.090	0.006	0.001	0.080	0.005	0.001
BMI	−0.029	0.013	0.281	−0.022	0.013	0.421	−0.011	0.013	0.685	−0.009	0.013	0.736			
Length of education				0.055	0.023	0.030	0.024	0.024	0.352	0.025	0.023	0.320			
Sustainable nutrition knowledge scores							0.134	0.020	<0.001	0.119	0.019	<0.001	0.125	0.019	<0.001
Environmentally responsible food preference scores										0.148	0.010	<0.001	0.148	0.010	<0.001
Adjusted *R*^2^	0.014			0.016			0.032			0.052			0.052		

Multiple linear regression.

## 4. Discussion

The MD is considered a sustainable diet model when regarded together with its socio-cultural, economic, and environmental benefits (21). In the present study, most of the individuals (52.3%) living in Turkey, a country with a coast on the Mediterranean, had a moderate level of adherence to the MD. Similarly, in studies conducted in Italy (22), Portugal (23) and different European countries (24) the most of individuals (59.4, 62.7 and 68.3%, respectively) adhered to the MD at a moderate level. On the other hand in a study conducted in Lithuania and Serbia, which are not Mediterranean countries, most of the participants (47.7%) had moderate levels of adherence but the rate of low levels of adherence was also high (39%) (25). These data emphasize that geography is important for individuals’ nutritional habits, food preferences, and diet quality. Although the availability of and accessibility to olive oil, legumes, whole grains, and fresh products (fruits, vegetables, and fish) are easier in Mediterranean countries, the low rate of individuals with high adherence to the MD in these studies is confusing. In a study comparing the trends of adherence to the MD at the global level over time, there was a significant decrease in the level of adherence to the MD between 1961 and 1965 and between 2000 and 2003 in many countries, including Turkey, but this decrease was smaller between 2004 and 2011 (26). This situation may have resulted from Westernization together with the changes in cultural, social, and political factors greatly impacting the changes in nutritional habits.

While the sociocultural level is one of the variables consistently associated with better adherence to the MD in the literature, it is controversial whether a higher education level is generally associated with adopting healthier diets and consuming healthier foods (27). Especially with Westernization, university students take an active role in the modern age. In a review, it is stated that the majority of university students show moderate adherence to the MD, and this is evident even among university students living in Mediterranean countries, especially those living far from their families (28). Same results were also found in studies evaluating the adherence of university students to the MD in Turkey (29, 30). Similarly, in the present study, in which most of the participants (78.1%) were university students/graduates, the adherence of individuals to the MD was moderate. It is thought that the changes in the food preferences of university students, their access to fresh food, the inadequacy of food preparation conditions due to their staying in dormitories and student houses, and negative financial conditions cause this situation.

The MD, which is described as sustainable, is associated with 4 criteria: 1. high consumption of vegetables and fruits, 2. low consumption of cheese, meat, and meat products, 3. low intake of sugar, sodium, total fat, saturated fat, and cholesterol, and 4. high consumption of fish and olive oil (13). A traditional MD includes the consumption of seasonal vegetables and fruits every day. In the present study, it is noteworthy that individuals of both sexes have levels lower than the recommendation (2 servings of vegetables/day, 3 servings of fruit/day) in terms of fruit and vegetable consumption. A study conducted by Marendić et al. (31) in Italy with a similar age group also determined that individuals’ adherence to vegetable and fruit consumption recommendations was very low, similar to our study. In another Italian study conducted with adults, it was also stated that adherence to the recommendations of fruit and vegetable consumption was low (32). These plant-derived foods, which have less environmental impact and high nutritional value, were consumed less than recommended may be an important problem in terms of sustainable nutrition.

The most characteristic feature of the MD is olive oil. In the present study, although most individuals with low, moderate, and high adherence to the MD indicated olive oil as the main culinary fat, compliance with the recommendation for olive oil amount was found low in both sexes. In a study conducted with Lebanese university students, the type of oil consumed by individuals, in general, was olive oil, but, similar to our study, the amount of olive oil consumed daily by the participants was below the recommendations (33). This may be due to the fact that olive oil is more expensive than other types of vegetable oil.

In the present study, legumes and nuts were the foods for which individuals’ adhered to the MD recommendations most. In particular, legume consumption among the men with high adherence to the MD is higher than among both the men and women with lower adherence. Nut consumption by individuals is compatible with the MD adherence level and is similar between the sexes. In a study conducted in Gulf countries (Saudi Arabia, Oman, and Kuwait), the highest consumption of legumes was seen in individuals with moderate adherence to the MD, and the highest consumption of nuts was determined in individuals with high adherence. However, in line with the recommendations, the rates of individuals consuming legumes and nuts were quite low (34). The fact that Turkey is located in geography rich in cereals and legumes may have caused a high level of adherence to these recommendations.

As an important food source, fish has various health benefits due to its antioxidant, anti-inflammatory, cardioprotective, and neuroprotective properties (35). However, the effect of seafood on a sustainable diet is not very clear in the literature. The health benefits of seafood consumption and the lower impact of fish consumption on greenhouse gas emissions compared to other animal-derived proteins are good points to be highlighted; however, the ecological impacts as a result of overfishing create a dilemma (36). Therefore, it is important to strengthen recommendations for consumption from accepted sustainable sources and species that are not overfished. Considering its effects on both health and the environment, the MD, which supports local and seasonal fish consumption, is seen as one of the healthiest nutritional patterns with low environmental impact (37). In the present study, although fish consumption by individuals with high adherence to the MD in both sexes is higher than in those with moderate and low adherence, it is noteworthy that only 37.3% of individuals with high adherence to the MD complied with the recommendations.

Age is associated with food choices, and it is stated that older individuals show higher adherence to the MD (27, 38). In our study, similar to the literature, a significant positive correlation was found between age and MEDAS score. Accordingly, it was determined that a one-unit increase in age increased the MD adherence score by 0.08 points. It is thought that this situation may be since elderly individuals behave more traditionally in their food preferences and that chronic diseases requiring nutrition therapy are seen more frequently with increasing age. On the other hand, because of changing living conditions and fast changes in their food preferences, younger people play a more active role in Westernization. In the present study, the majority of participants (72.1%) were aged between 18 and 29. Therefore, future studies with larger samples in the older age group would be a better guide for evaluating this relationship.

As a sustainable dietary pattern, the MD gains importance not only with its nutritional recommendations but also with the protection of biodiversity, local production, and the culture of each community. It has been shown that there is a relationship between the health-promoting and eco-friendly behaviors of individuals and their adherence to the MD (39). Further, it is also known that there is a relationship between the level of nutrition knowledge, which is influential in improving health, and the adherence of individuals to the MD (40). In the present study, individuals with a higher level of knowledge about sustainable nutrition adopted a more sustainable nutrition model. In fact, an increase of one unit in the sustainable nutrition knowledge score increases the MD adherence score by 0.125 points. According to the MD model, plant-derived foods, which are recommended to be consumed more frequently in order to provide a healthy diet, also have less impact on the environment during production and consumption (39).

Eco-friendly approaches such as organic agriculture and the MD are seen as promising models for sustainable diets (41). In the present study was found that environmentally responsible food choices were a factor affecting adherence to the MD and this result is in agreement with the literature. Individuals with high adherence to the MD stated that they preferred to consume environmentally labeled foods more than those with moderate or low adherence. As a result of the study conducted by Yardimci and Demirer (42) to examine the relationship between the level of adherence to the Mediterranean diet and the awareness of the Ecological Footprint of Turkish adults, it was determined that as individuals’ adherence to the Mediterranean diet increases, their awareness of ecological footprints will also increase. In the literature, it is stated that the use of eco-labels for sustainable food shopping may be beneficial for consumers with a preference for sustainable nutrition but without sufficient knowledge. The use of eco-labels in food shopping affects positively the organic food choices of individuals and becomes a source of motivation to choose these foods (43).

One of the factors evaluated within the scope of environmentally responsible food choices was GMOs in our study. It was determined that individuals with high adherence to the MD avoided consuming food with GMOs more than those with moderate or low adherence (*p* < 0.001). Although there is concern about the possible harmful effects of foods with GMOs on the environment, when evaluated in terms of sustainability it is stated that the correct use of GMOs technology may be important in achieving the goal of preventing global hunger. More studies are needed to determine the economic, environmental, and health-related indirect effects of foods with GMOs (44).

In addition, the role of the MD in strengthening sustainable food systems through regional development strategies and traditional local products is emphasized (45). Regarding its environmental impact, the MD supports the consumption of local and seasonal products (11). It is recommended to consume more seasonal and locally produced foods in order to reduce the energy inputs and greenhouse gas emissions caused by household food consumption (46). In our study, individuals with high adherence to the MD preferred to buy milk and dairy products from local markets and they preferred imported foods less. In a study with Italian families, households with high adherence to the MD were more likely to purchase both organic and local products, while an increase in household size decreased the likelihood of purchasing local products (41).

The adherence of individuals to the MD was evaluated with MEDAS, which is an internationally valid and reliable scale, and the evaluation of adherence to the MD from both a nutritional and sustainable perspective constitutes the strength of the present study. Since there is no valid and reliable tool developed to evaluate the sustainable nutrition knowledge level in the international literature, the use of statements prepared in line with the existing literature for this purpose is one of the limitations of our study. Another limitation is that although the sample size was high, most of the participants consisted of young adults and individuals with a high level of education. It is thought that this may have been since participation in online studies is easier for this age group. For the sample to represent the universe, it is recommended to ensure a homogeneous distribution of different age groups for future research. Evaluation of weight and height according to the declaration is also among the limitations of this study.

As a result, investigating individuals’ diet quality and their perceptions of food preferences is a valuable approach to appropriately shifting dietary behavior in the desired direction and identifying effective strategies. In the present study, the factors that may affect adherence to the MD were examined by asking questions about the sustainable nutritional knowledge and environmentally responsible food choices of individuals. It was found that some changes are needed in the dietary habits of Turkish adults to meet the nutritional recommendations and environmental guidelines for sustainability. Since nutritious foods such as fresh vegetables and fruits, which are an important component of the MD, are consumed less than recommended, multidimensional approaches should be developed both to increase individuals’ awareness of the importance of fruit and vegetable consumption and to provide access to these foods. Insufficient fish consumption is an important problem in terms of both healthy nutrition and the environmental effects of nutrition. In order to increase fish consumption among individuals, not only ensuring adequate access to fish but also increasing awareness of cultural adoption and the possible benefits of fish consumption is important. The development of policies that will increase adherence to the MD is extremely important in terms of its contribution to the health of the public as well as to the protection of the world. For this purpose, it is necessary to encourage nutritionally adequate, sustainable, and eco-friendly nutritional behaviors in terms of nutrition. To promote sustainable nutrition, determining the knowledge level of individuals concerning sustainable nutrition is very important, so there is a need for an internationally valid sustainable nutrition knowledge assessment tool.

## Data availability statement

The original contributions presented in the study are included in the article/Supplementary material, further inquiries can be directed to the corresponding author.

## Ethics statement

The studies involving human participants were reviewed and approved by Gazi University Ethics Committee. The patients/participants provided their written informed consent to participate in this study.

## Author contributions

EY contributed to the conception, design of the study, and wrote the first draft of the manuscript. HB organized the database and performed the statistical analysis. EY and HB wrote sections of the manuscript. All authors contributed to the article and approved the submitted version.

## Conflict of interest

The authors declare that the research was conducted in the absence of any commercial or financial relationships that could be construed as a potential conflict of interest.

## Publisher’s note

All claims expressed in this article are solely those of the authors and do not necessarily represent those of their affiliated organizations, or those of the publisher, the editors and the reviewers. Any product that may be evaluated in this article, or claim that may be made by its manufacturer, is not guaranteed or endorsed by the publisher.

## Supplementary material

The Supplementary material for this article can be found online at: https://www.frontiersin.org/articles/10.3389/fnut.2023.1158155/full#supplementary-material

## References

[1] Di Marco M Baker ML Daszak P De Barro P Eskew EA Godde CM. Sustainable development must account for pandemic risk. Proc Natl Acad Sci. (2020) 117:3888–92. doi: 10.1073/pnas.200165511, PMID: 32060123 PMC7049118

[2] Butlin J. Our common future. By world commission on environment and development. (London, Oxford university press, 1987, pp.383). J Int Dev. (1989) 1:284–7. doi: 10.1002/jid.3380010208

[3] Grosso G Mateop A Rangelov N Buzeti T Birt C. Nutrition in the context of the sustainable development goals. Eur J Pub Health. (2020) 30:i19–23. doi: 10.1093/eurpub/ckaa034, PMID: 32391903 PMC7213573

[4] David T Clark M. Global diets link environmental sustainability and human health. Nature. (2014) 515:518–22. doi: 10.1038/nature13959, PMID: 25383533

[5] Coats L Aboul-Enein BH Dodge E Benajiba N Kruk J Khaled MB. Perspectives of environmental health promotion and the mediterranean diet: a thematic narrative synthesis. J Hunger Environ Nutr. (2022) 17:85–107. doi: 10.1080/19320248.2020.1777242

[6] Serra-Majem L Tomaino L Dernini S Berry EM Lairon D de la Cruz N. Updating the mediterranean diet pyramid towards sustainability: focus on environmental concerns. Int J Environ Res Public Health. (2020) 17:8758. doi: 10.3390/ijerph17238758, PMID: 33255721 PMC7728084

[7] Fanzo J Bellows AL Spiker ML Thorne-Lyman AL Bloem MW. The importance of food systems and the environment for nutrition. Am J Clin Nutr. (2021) 113:7–16. doi: 10.1093/ajcn/nqaa313, PMID: 33236086 PMC7717136

[8] Johnston JL Fanzo JC Cogill B. Understanding sustainable diets: a descriptive analysis of the determinants and processes that influence diets and their impact on health, food security, and environmental sustainability. Adv Nutr. (2014) 5:418–29. doi: 10.3945/an.113.005553, PMID: 25022991 PMC4085190

[9] Barbara B Dernini S. Sustainable diets: the Mediterranean diet as an example. Public Health Nutr. (2011) 14:2285–7. doi: 10.1017/S1368980011002527, PMID: 22166185

[10] Corné VD Aiking H. Defining a nutritionally healthy, environmentally friendly, and culturally acceptable low lands diet. Int J Life Cycle Assess. (2016) 21:688–700. doi: 10.1007/s11367-015-1007-3

[11] Dernini S Berry EM Serra-Majem L La Vecchia C Capone R Medina FX. Med diet 4.0: the Mediterranean diet with four sustainable benefits. Public Health Nutr. (2017) 20:1322–30. doi: 10.1017/S1368980016003177, PMID: 28003037 PMC10261651

[12] Pekcan AG. Sustainable diet and nutrition pattern: plant-based nutrition. J Nutr Diet. (2019) 47:1–10. doi: 10.33076/2019.BDD.1268

[13] Bayram SŞ Aktaş N. Mediterranean diet and frequently used indexes for measuring Mediterranean diet quality. International Eurasian congress on "natural nutrition and healthy life' 12-15 July (2018). In Proceedings book. M. R. Karaman, N. Artık, N. Şanlıer (Eds.). Ankara University Institute of Food Safety "Pelin Ofset; Press”.

[14] Sáez-Almendros S Obrador B Bach-Faig A Serra-Majem L. Environmental footprints of Mediterranean versus Western dietary patterns: beyond the health benefits of the Mediterranean diet. Environ Health. (2013) 12:1–8. doi: 10.1186/1476-069X-12-118, PMID: 24378069 PMC3895675

[15] Trajkovska Petkoska A Trajkovska-Broach A. Mediterranean diet: a nutrient-packed diet and a healthy lifestyle for a sustainable world. J Sci Food Agric. (2021) 101:2627–33. doi: 10.1002/jsfa.10907, PMID: 33140412

[16] Smit ES Meijers MHC van der Laan LN. Using virtual reality to stimulate healthy and environmentally friendly food consumption among children: an interview study. Int J Environ Res Public Health. (2021) 18:1088. doi: 10.3390/ijerph18031088, PMID: 33530495 PMC7908483

[17] Başar Ş Başar EE. How does the environmental knowledge of Turkish households affect their environmentally responsible food choices? The mediating effects of environmental concerns. Int J Agric Environ Food Sci. (2020) 4:348–55. doi: 10.31015/jaefs.2020.3.14

[18] Martínez-Gonzáleze MÁ Corella D Salas-Salvadó J Ros E Covas MI Fiol M. Cohort profile: design and methods of the PREDIMED study. Int J Epidemiol. (2012) 41:377–85. doi: 10.1093/ije/dyq250, PMID: 21172932

[19] Pehlivanoğlu EFÖ Balcıoğlu H Ünlüoğlu İ. The validity and reliability of the adaptation of the Mediterranean diet adherence scale to Turkish. Osmangazi Med J. (2020) 42:160–4. doi: 10.20515/otd.504188

[20] Gülsöz S. The evaluation of the levels of knowledge and practice on sustainable nutrition of individuals’ aged twenty years and over In: . Health Sciences Institute, Nutrition And Dietetics. ed. Aksoydan E. (Ankara: Başkent University) (2017)

[21] Yüksel A Özkul E. Evaluation of sustainable dietary models. Bursa Uludağ Univ J Faculty Agric. (2021) 35:467–81.

[22] Biasini B Rosi A Menozzi D Scazzina F. Adherence to the Mediterranean diet in association with self-perception of diet sustainability, anthropometric and sociodemographic factors: a cross-sectional study in Italian adults. Nutrients. (2021) 13:3282. doi: 10.3390/nu13093282, PMID: 34579159 PMC8468784

[23] Andrade V Jorge R García-Conesa MT Philippou E Massarou M Chervenkov M. Mediterranean diet adherence and subjective well-being in a sample of Portuguese adults. Nutrients. (2020) 12:3837. doi: 10.3390/nu12123837, PMID: 33339084 PMC7765516

[24] Quarta S Massaro M Chervenkov M Ivanova T Dimitrova D Jorge R. Persistent moderate-to-weak Mediterranean diet adherence and low scoring for plant-based foods across several southern European countries: are we overlooking the Mediterranean diet recommendations? Nutrients. (2021) 13:1432. doi: 10.3390/nu13051432, PMID: 33922771 PMC8145023

[25] Novak D Štefan L Prosoli R Emeljanovas A Mieziene B Milanović I. Mediterranean diet and its correlates among adolescents in non-Mediterranean European countries: a population-based study. Nutrients. (2017) 9:177. doi: 10.3390/nu9020177, PMID: 28241432 PMC5331608

[26] Vilarnau C Stracker DM Funtikov A da Silva R Estruch R Bach-Faig A. Worldwide adherence to Mediterranean diet between 1960 and 2011. Eur J Clin Nutr. (2019) 72:83–91. doi: 10.1038/s41430-018-0313-9, PMID: 30487566

[27] Buscemi S. What are the determinants of adherence to the mediterranean diet? Int J Food Sci Nutr. (2021) 72:143–4. doi: 10.1080/09637486.2021.1889995, PMID: 33685338

[28] Antonopoulou M Mantzorou M Serdari A Bonotis K Vasios G Pavlidou E. Evaluating Mediterranean diet adherence in university student populations: does this dietary pattern affect students' academic performance and mental health? Int J Health Plann Manag. (2020) 35:5–21. doi: 10.1002/hpm.2881, PMID: 31514237

[29] Sönmez T. Determination of university students' adaptation to Mediterranean diet and nutritional status. J Health Life Sci. (2021) 3:85–90. doi: 10.33308/2687248X.202131209

[30] Baydemir C Ozgur EG Balci S. Evaluation of adherence to Mediterranean diet in medical students at Kocaeli University. Turkey J Int Med Res. (2018) 46:1585–94. doi: 10.1177/0300060518757, PMID: 29444610 PMC6091843

[31] Marendić M Polić N Matek H Oršulić L Polašek O Kolčić I. Mediterranean diet assessment challenges: validation of the Croatian version of the 14-item Mediterranean diet serving score (MDSS) questionnaire. PLoS One. (2021) 16:e0247269. doi: 10.1371/journal.pone.0247269, PMID: 33647026 PMC7920370

[32] Caparello G Galluccio A Giordano C Lofaro D Barone I Morelli C. Adherence to the Mediterranean diet pattern among university staff: a cross-sectional web-based epidemiological study in southern Italy. Int J Food Sci Nutr. (2020) 71:581–92. doi: 10.3390/nu13041264, PMID: 31690142

[33] Karam J Bibiloni MDM Serhan M Tur JA. Adherence to Mediterranean diet among Lebanese university students. Nutrients. (2021) 13:1264. doi: 10.3390/nu13041264, PMID: 33921397 PMC8069129

[34] Shatwan IM Alhinai EA Alawadhi B Surendran S Aljefree NM Almoraie NM. High adherence to the Mediterranean diet is associated with a reduced risk of obesity among adults in gulf countries. Nutrients. (2021) 13:995. doi: 10.3390/nu13030995, PMID: 33808684 PMC8003341

[35] Chen J Jayachandran M Bai W Xu B. A critical review on the health benefits of fish consumption and its bioactive constituents. Food Chem. (2022) 369:130874. doi: 10.1016/j.foodchem.2021.130874, PMID: 34455321

[36] Lofstedt A de Roos B Fernandes PG. Less than half of the European dietary recommendations for fish consumption are satisfied by national seafood supplies. Eur J Nutr. (2021) 60:4219–28. doi: 10.1007/s00394-021-02580-6, PMID: 33999272 PMC8572203

[37] Fresán U Martínez-Gonzalez MA Sabaté J Bes-Rastrollo M. The Mediterranean diet, an environmentally friendly option: evidence from the Seguimiento Universidad de Navarra (SUN) cohort. Public Health Nutr. (2018) 21:1573–82. doi: 10.1017/S1368980017003986, PMID: 29380717 PMC10261578

[38] Kyprianidou M Christophi CA Giannakou K. Quarantine during COVID-19 outbreak: adherence to the Mediterranean diet among the Cypriot population. Nutrition. (2021) 90:111313. doi: 10.1016/j.nut.2021.111313, PMID: 34119718 PMC9759705

[39] Cavaliere A De Marchi E Banterle A. Exploring the adherence to the Mediterranean diet and its relationship with individual lifestyle: the role of healthy behaviors, pro-environmental behaviors, income, and education. Nutrients. (2018) 10:141. doi: 10.3390/nu10020141, PMID: 29382086 PMC5852717

[40] Bottcher MR Marincic PZ Nahay KL Baerlocher BE Willis AW Park J. Nutrition knowledge and Mediterranean diet adherence in the Southeast United States: validation of a field-based survey instrument. Appetite. (2017) 111:166–76. doi: 10.1016/j.appet.2016.12.029, PMID: 28017910

[41] Annunziata A Agovino M Mariani A. Sustainability of Italian families' food practices: Mediterranean diet adherence combined with organic and local food consumption. J Clean Prod. (2019) 206:86–96. doi: 10.1016/j.jclepro.2018.09.155

[42] Yardimci H Demirer B. Is high adaptation to the Mediterranean diet effective in increasing ecological footprint awareness? A cross-sectional study from Turkey. J Sci Food Agric. (2022) 102:3724–9. doi: 10.1002/jsfa.11720, PMID: 34907547

[43] Thangarajan H Remesh SD Kumar PS Chandran ARR Rajaendran S Sandrasaigaran P. Genetically modified foods for sustainable food security: debunking the myths. Malaysian journal of science and advanced. Technology. (2021):129–35. doi: 10.56532/mjsat.v1i4.28

[44] Dernini S Berry EM. Mediterranean diet: from a healthy diet to a sustainable dietary pattern. Front Nutr. (2015) 2:15. doi: 10.3389/fnut.2015.00015, PMID: 26284249 PMC4518218

[45] Meybeck A Burlıngame B Lacırıgnola C El Bılalı H Philippe DEBS Capone R. Developing a methodological approach for assessing the sustainability of diets: the Mediterranean diet as a case study. New Medit: Mediterranean journal of economics, agriculture and environment = revue Méditerranéenne d′Economie agriculture et. Environment. (2013) 12:28.

[46] Neumayr L Moosauer C. How to induce sales of sustainable and organic food: the case of a traffic light eco-label in online grocery shopping. J Clean Prod. (2021) 328:129584. doi: 10.1016/j.jclepro.2021.129584

