# Supplementary material for: Evaluation of adherence to the Mediterranean diet with sustainable nutrition knowledge and environmentally responsible food choices
Source: Front Nutr. 2023 Apr 12;10:1158155. doi: 10.3389/fnut.2023.1158155 (PMC10130392; doi:10.3389/fnut.2023.1158155)
Supplement: Supplementary file 1 [file Table_1.DOCX]

Supplementary Material

Evaluation of Adherence to the Mediterranean Diet with Sustainable Nutrition Knowledge and Environmentally Responsible Food Choices

Emine Yassıbaş^*^, Hatice Bölükbaşı

*** Correspondence:** Emine Yassıbaş: [eyassibas@gazi.edu.tr](mailto:eyassibas@gazi.edu.tr)

# Supplementary Tables

**Suppl Table 1. Distribution of participants' responses to sustainable nutrition knowledge questions according to sex and Mediterranean Diet adherence**

| **Sustainable nutrition knowledge questions** | **Total** | | | | | | **Men** | | | | | | **Women** | | | | | | **p** |
| --- | --- | --- | --- | --- | --- | --- | --- | --- | --- | --- | --- | --- | --- | --- | --- | --- | --- | --- | --- |
|  | **Low** | | **Moderate** | | **High** | | **Low** | | **Moderate** | | **High** | | **Low** | | **Moderate** | | **High** | |  |
|  | **n** | **%** | **n** | **%** | **n** | **%** | **n** | **%** | **n** | **%** | **n** | **%** | **n** | **%** | **n** | **%** | **n** | **%** |  |
| **Do you think sustainable nutrition should be economical?** | | | | | | | | | | | | | | | | | | | |
| **Yes** | 358^a^ | 83,9 | 811^b^ | 89,6 | 352^a,b^ | 88,2 | 130^a^ | 76,5 | 272^b^ | 86,6 | 107^a,b^ | 81,6 | 228^a^ | 88,7 | 539^a^ | 91,0 | 254^a^ | 91,5 |  |
| **No** | 19^a^ | 4,4 | 33^a^ | 3,6 | 15^a^ | 3,8 | 15^a^ | 8,8 | 17^a^ | 5,4 | 9^a^ | 6,9 | 4^a^ | 1,6 | 16^a^ | 2,7 | 6^a^ | 2,2 |  |
| **No idea** | 50^a^ | 11,7 | 62^b^ | 6,8 | 32^a,b^ | 8,0 | 25^a^ | 14,7 | 25^b^ | 8,0 | 15^a,b^ | 11,5 | 25^a^ | 9,7 | 37^a^ | 6,3 | 17^a^ | 6,3 |  |
|  | **p=0,042** | | | | | | p=0,085 | | | | | | p=0,344 | | | | | | **p=0,000** |
| **Do you think sustainable nutrition is expensive?** | | | | | | | | | | | | | | | | | | | |
| **Yes** | 138^a^ | 32,3 | 314^a^ | 34,7 | 129^a^ | 32,3 | 48^a^ | 28,2 | 122^b^ | 38,9 | 46^a^,^b^ | 35,1 | 90^a^ | 35,0 | 192^a^ | 32,4 | 83^a^ | 31,0 |  |
| **No** | 168a | 39,4 | 378^a^ | 41,7 | 167^a^ | 41,9 | 81^a^ | 47,7 | 130^a^ | 41,4 | 48^a^ | 36,7 | 87^a^ | 33,9 | 248^b^ | 41,9 | 119^b^ | 44,4 |  |
| **No idea** | 121^a^ | 28,3 | 214^a^ | 23,6 | 103^a^ | 25,8 | 41^a^,^b^ | 24,1 | 62^b^ | 19,7 | 37^a^ | 28,2 | 80^a^ | 31,1 | 152^a^ | 25,7 | 66^a^ | 24,6 |  |
|  | p=0,438 | | | | | | p=0,064 | | | | | | p=0,120 | | | | | | p=0,190 |
| **Do you think that consumption of fish instead of red meat contributes to sustainable nutrition?** | | | | | | | | | | | | | | | | | | | |
| **Yes** | 177^a^ | 41,5 | 483^b^ | 53,3 | 234^b^ | 58,6 | 68^a^ | 40,0 | 162^b^ | 51,6 | 71^b^ | 54,2 | 109^a^ | 42,4 | 321^b^ | 54,2 | 163^b^ | 60,8 |  |
| **No** | 114^a^ | 26,7 | 212^a^ | 23,4 | 69^b^ | 17,3 | 50^a^ | 29,4 | 85^a^,^b^ | 27,1 | 24^b^ | 18,3 | 64^a^ | 24,9 | 127^a^,^b^ | 21,5 | 45^b^ | 16,8 |  |
| **No idea** | 136^a^ | 31,8 | 211^b^ | 23,3 | 96^b^ | 21,1 | 52^a^ | 30,6 | 67^b^ | 21,3 | 36^a^,^b^ | 27,5 | 84^a^ | 32,7 | 144^b^ | 24,3 | 60^b^ | 22,4 |  |
|  | **p=0,000** | | | | | | **p=0,018** | | | | | | **p=0,001** | | | | | | p=0,072 |
| **Do you think that in sustainable nutrition, foods should be consumed in season?** | | | | | | | | | | | | | | | | | | | |
| **Yes** | 388^a^ | 90,9 | 839^a^ | 92,6 | 361^a^ | 90,5 | 155^a^ | 91,1 | 281^a^ | 89,5 | 110^a^ | 84,0 | 233^a^ | 90,6 | 558^a^ | 94,3 | 251^a^ | 93,7 |  |
| **No** | 7^a^ | 1,6 | 27^a^ | 3,0 | 12^a^ | 3,0 | 4^a^ | 2,4 | 15^a^ | 4,8 | 6^a^ | 4,6 | 3^a^ | 1,2 | 12^a^ | 2,0 | 6^a^ | 2,2 |  |
| **No idea** | 32^a^ | 7,5 | 40^b^ | 4,4 | 26^a^,^b^ | 6,5 | 11^a^,^b^ | 6,5 | 18^b^ | 5,7 | 15^a^ | 11,5 | 21^a^ | 8,2 | 22^b^ | 3,7 | 11^a^,^b^ | 4,1 |  |
|  | p=0,094 | | | | | | p=0,162 | | | | | | p=0,062 | | | | | | **p=0,003** |
| **Do you think there is a relationship between the consumption of foods imported from abroad and sustainable nutrition?** | | | | | | | | | | | | | | | | | | | |
| **Yes** | 116^a^ | 27,2 | 282^a^ | 31,1 | 121^a^ | 30,3 | 48^a^ | 28,2 | 93^a^ | 29,6 | 39^a^ | 29,8 | 68^a^ | 26,5 | 189^a^ | 31,9 | 82^a^ | 30,6 |  |
| **No** | 155^a^ | 36,3 | 326^a^ | 36,0 | 151^a^ | 37,9 | 69^a^ | 40,6 | 133^a^ | 42,4 | 51^a^ | 38,9 | 86^a^ | 33,5 | 193^a^ | 32,6 | 100^a^ | 37,3 |  |
| **No idea** | 156^a^ | 36,5 | 298^a^ | 32,9 | 127^a^ | 31,8 | 53^a^ | 31,2 | 88^a^ | 28,0 | 41^a^ | 31,3 | 103^a^ | 40,0 | 210^a^ | 35,5 | 86^a^ | 32,1 |  |
|  | p=0,491 | | | | | | p=0,924 | | | | | | p=0,252 | | | | | | **p=0,006** |
| **Do you think reducing food packaging is important in environmentally friendly nutrition?** | | | | | | | | | | | | | | | | | | | |
| **Yes** | 337^a^ | 78,9 | 767^b^ | 84,7 | 343^b^ | 86,0 | 119^a^ | 70,0 | 248^b^ | 79,0 | 104^a^,^b^ | 79,4 | 218^a^ | 84,8 | 519^a^ | 87,7 | 239^a^ | 89,2 |  |
| **No** | 40^a^ | 9,4 | 78^a^ | 8,6 | 27^a^ | 6,7 | 22^a^ | 12,9 | 44^a^ | 14,0 | 13^a^ | 9,9 | 18^a^ | 7,0 | 34^a^ | 5,7 | 14^a^ | 5,2 |  |
| **No idea** | 50^a^ | 11,7 | 61^b^ | 6,7 | 29^b^ | 7,3 | 29^a^ | 17,1 | 22^b^ | 7,0 | 14^a^,^b^ | 10,7 | 21^a^ | 8,2 | 39^a^ | 6,6 | 15^a^ | 5,6 |  |
|  | **p=0,013** | | | | | | **p=0,011** | | | | | | p=0,666 | | | | | | **p=0,000** |
| **Do you think that consuming foods by freezing contributes to sustainable nutrition?** | | | | | | | | | | | | | | | | | | | |
| **Yes** | 175^a^ | 41,0 | 424^b^ | 46,8 | 195^b^ | 48,9 | 74^a^ | 43,5 | 139^a^ | 44,3 | 51^a^ | 39,0 | 101^a^ | 39,3 | 285^b^ | 48,1 | 144^b^ | 53,7 |  |
| **No** | 162^a^ | 37,9 | 342^a^ | 37,7 | 139^a^ | 34,8 | 61^a^ | 35,9 | 138^a^ | 43,9 | 56^a^ | 42,7 | 101^a^ | 39,3 | 204^a^,^b^ | 34,5 | 83^b^ | 31,0 |  |
| **No idea** | 90^a^ | 21,1 | 140^b^ | 15,5 | 65^a^,^b^ | 16,3 | 35^a^ | 20,6 | 37^b^ | 11,8 | 24^a^,^b^ | 18,3 | 55^a^ | 21,4 | 103^a^ | 17,4 | 41^a^ | 15,3 |  |
|  | p=0,050 | | | | | | p=0,069 | | | | | | **p=0,022** | | | | | | **p=0,021** |
| **Do you think that not consuming sugary drinks, carbonated, high-calorie drinks contributes to sustainable nutrition?** | | | | | | | | | | | | | | | | | | | |
| **Yes** | 228^a^ | 53,4 | 492^a^ | 54,3 | 237^a^ | 59,4 | 82^a^,^b^ | 48,2 | 133^b^ | 42,4 | 71^a^ | 54,2 | 146^a^ | 56,8 | 359^a^ | 60,6 | 166^a^ | 62,0 |  |
| **No** | 153^a^ | 35,8 | 338^a^ | 37,3 | 130^a^ | 32,6 | 68^a^,^b^ | 40,0 | 147^b^ | 46,8 | 45^a^ | 34,4 | 85^a^ | 33,1 | 191^a^ | 32,3 | 85^a^ | 31,7 |  |
| **No idea** | 46^a^ | 10,8 | 76^a^ | 8,4 | 32^a^ | 8,0 | 20^a^ | 11,8 | 34^a^ | 10,8 | 15^a^ | 11,5 | 26^a^ | 10,1 | 42^a^ | 7,1 | 17^a^ | 6,3 |  |
|  | p=0,224 | | | | | | p=0,152 | | | | | | p=0,461 | | | | | | **p=0,000** |
| **Do you think that consuming more local foods can contribute to sustainable nutrition?** | | | | | | | | | | | | | | | | | | | |
| **Yes** | 294^a^ | 68,8 | 627^a^ | 69,2 | 292^a^ | 73,2 | 120^a^ | 70,6 | 225^a^ | 71,6 | 93^a^ | 71,0 | 174^a^ | 67,7 | 6402^a^ | 67,9 | 199^a^ | 74,2 |  |
| **No** | 73^a^ | 17,1 | 171^a^ | 18,9 | 59^a^ | 14,8 | 30^a^ | 17,6 | 53^a^ | 16,9 | 20^a^ | 15,3 | 43^a^ | 16,7 | 118^a^ | 19,9 | 39^a^ | 14,6 |  |
| **No idea** | 60^a^ | 14,1 | 108^a^ | 11,9 | 48^a^ | 12,0 | 20^a^ | 11,8 | 36^a^ | 11,5 | 18^a^ | 13,7 | 40^a^ | 15,6 | 72^a^ | 12,2 | 30^a^ | 11,2 |  |
|  | p=0,334 | | | | | | p=0,953 | | | | | | p=0,152 | | | | | | p=0,725 |
| **Do you think that not consuming processed foods can contribute to sustainable nutrition?** | | | | | | | | | | | | | | | | | | | |
| **Yes** | 190^a^ | 44,5 | 457^b^ | 50,4 | 230^c^ | 57,6 | 62^a^ | 36,5 | 135^a^ | 43,0 | 72^b^ | 55,0 | 128^a^ | 49,8 | 322^a^,^b^ | 54,4 | 158^b^ | 59,0 |  |
| **No** | 154^a^ | 36,1 | 333^a^ | 36,8 | 116^b^ | 29,1 | 62^a^,^b^ | 36,5 | 134^b^ | 42,7 | 36^a^ | 27,5 | 92^a^ | 35,8 | 199^a^ | 33,6 | 80^a^ | 29,8 |  |
| **No idea** | 83^a^ | 19,4 | 116^b^ | 12,8 | 53^b^ | 13,3 | 46^a^ | 46^a^ | 45^b^ | 14,3 | 23^a^,^b^ | 17,5 | 37^a^ | 14,4 | 71^a^ | 12,0 | 30^a^ | 11,2 |  |
|  | **p=0,000** | | | | | | **p=0,000** | | | | | | p=0,320 | | | | | | **p=0,000** |
| **Sustainable nutrition definition** | | | | | | | | | | | | | | | | | | | |
| **True** | 328^a^ | 76,8 | 742^a^ | 81,9 | 319^a^ | 79,9 | 129^a^ | 75,9 | 246^a^ | 78,3 | 95^a^ | 72,5 | 199^a^ | 77,4 | 496^a^ | 83,8 | 224^a^ | 83,6 |  |
| **False** | 99^a^ | 23,2 | 164^a^ | 18,1 | 80^a^ | 20,1 | 41^a^ | 24,1 | 68^a^ | 21,7 | 36^a^ | 27,5 | 58^a^ | 22,6 | 96^a^ | 16,2 | 44^a^ | 16,4 |  |
|  | p=0,093 | | | | | | p=0,411 | | | | | | p=0,068 | | | | | | **p=0,003** |
| **Sustainable diet definition** | | | | | | | | | | | | | | | | | | | |
| **True** | 381^a^ | 89,2 | 847^b^ | 93,5 | 373^a,b^ | 93,5 | 149^a^ | 87,6 | 284^a^ | 90,4 | 117^a^ | 89,3 | 232^a^ | 90,3 | 563^b^ | 95,1 | 256^a,b^ | 95,5 |  |
| **False** | 46^a^ | 10,8 | 59^b^ | 6,5 | 26^a^,^b^ | 6,5 | 21^a^ | 12,4 | 30^a^ | 9,6 | 14^a^ | 10,7 | 25^a^ | 9,7 | 29^b^ | 4,9 | 12^a,b^ | 4,5 |  |
|  | **p=0,015** | | | | | | p=0,632 | | | | | | **p=0,012** | | | | | | **p=0,000** |
| **Do you think that the production of food causes greenhouse gas increase and water pollution?** | | | | | | | | | | | | | | | | | | | |
| **Yes** | 233^a^ | 54,5 | 508^a^ | 56,0 | 223^a^ | 55,9 | 77^a^ | 45,3 | 171^a^ | 54,5 | 63^a^ | 48,1 | 156^a^ | 60,7 | 337^a^ | 56,9 | 160^a^ | 59,7 |  |
| **No** | 87^a^ | 20,4 | 180^a^ | 19,9 | 93^a^ | 23,3 | 44^a^ | 25,9 | 82^a^ | 26,1 | 41^a^ | 31,3 | 43^a^ | 16,7 | 98^a^ | 16,6 | 52^a^ | 19,4 |  |
| **No idea** | 107^a^ | 25,1 | 218^a^ | 24,1 | 83^a^ | 20,8 | 49^a^ | 28,8 | 61^a^ | 19,4 | 27^a^ | 20,6 | 58^a^ | 22,6 | 157^a^ | 26,5 | 56^a^ | 20,9 |  |
|  | p=0,472 | | | | | | p=0,105 | | | | | | p=0,373 | | | | | | **p=0,000** |
| **If so, which food group do you think has the least environmental impact?** | | | | | | | | | | | | | | | | | | | |
| **Meat and meat products** | 38^a^ | 8,9 | 93^a^ | 10,3 | 44^a^ | 11,0 | 18^a^ | 10,6 | 38^a^ | 12,1 | 16^a^ | 12,2 | 20^a^ | 7,8 | 55^a^ | 9,3 | 28^a^ | 10,4 |  |
| **Milk and milk products** | 41^a^ | 9,6 | 75^a^ | 8,3 | 41^a^ | 10,3 | 12^a^ | 7,1 | 27^a^ | 8,6 | 16^a^ | 12,2 | 29^a^ | 11,3 | 48^a^ | 8,1 | 25^a^ | 9,3 |  |
| **Fruit and vegetables** | 147^a^ | 34,4 | 355^a^ | 39,2 | 158^a^ | 39,6 | 46^a^ | 27,1 | 121^b^ | 38,5 | 43^a,b^ | 32,8 | 101^a^ | 39,3 | 234^a^ | 39,5 | 115^a^ | 42,9 |  |
| **Bread and similar products** | 37^a^ | 8,7 | 79^a^ | 8,7 | 30^a^ | 7,5 | 20^a^ | 11,8 | 28^a^ | 8,9 | 14^a^ | 10,7 | 17^a^ | 6,6 | 51^a^ | 8,6 | 16^a^ | 6,0 |  |
| **No idea** | 164^a^ | 38,4 | 304^a^ | 33,6 | 126^a^ | 31,6 | 74^a^ | 43,5 | 100^b^ | 31,8 | 42^a^,^b^ | 32,1 | 90^a^ | 35,0 | 204^a^ | 34,5 | 84^a^ | 31,3 |  |
|  | p=0,432 | | | | | | p=0,128 | | | | | | p=0,607 | | | | | | **p=0,047** |
| **Which of the following foods do you think contributes more to greenhouse gases?** | | | | | | | | | | | | | | | | | | | |
| **Bread** | 57^a^ | 13,3 | 133^a^ | 14,7 | 60^a^ | 15,0 | 19^a^ | 11,2 | 47^a^ | 15,0 | 22^a^ | 16,8 | 38^a^ | 14,8 | 86^a^ | 14,5 | 38^a^ | 14,2 |  |
| **Chicken** | 22^a^ | 5,2 | 47^a^ | 5,2 | 18^a^ | 4,5 | 8^a^ | 4,7 | 21^a^ | 6,7 | 13^a^ | 9,9 | 14^a^ | 5,4 | 26^a^ | 4,4 | 5^a^ | 1,9 |  |
| **Rice** | 21^a^ | 4,9 | 44^a^ | 4,9 | 12^a^ | 3,0 | 6^a^ | 3,5 | 14^a^ | 4,5 | 1^a^ | 0,8 | 15^a^ | 5,8 | 30^a^ | 5,1 | 11^a^ | 4,1 |  |
| **Steak** | 82^a^ | 19,2 | 179^a^ | 19,8 | 113^b^ | 28,3 | 29^a^ | 17,1 | 56^a^ | 17,8 | 34^a^ | 26,0 | 53^a^,^b^ | 20,6 | 123^b^ | 20,8 | 79^a^ | 29,5 |  |
| **Milk** | 11^a^ | 2,6 | 59^b^ | 6,5 | 16^a^,^b^ | 4,0 | 8^a^ | 4,7 | 26^a^ | 8,3 | 9^a^ | 6,9 | 3^a^ | 1,2 | 33^b^ | 5,6 | 7^a^,^b^ | 2,6 |  |
| **No idea** | 234^a^ | 54,8 | 444^a^,^b^ | 49,0 | 180^b^ | 45,1 | 100^a^ | 58,8 | 150^a^,^b^ | 47,8 | 52^b^ | 39,7 | 134^a^ | 52,1 | 294^a^ | 49,7 | 128^a^ | 47,8 |  |
|  | **p=0,002** | | | | | | **p=0,029** | | | | | | **p=0,010** | | | | | | **p=0,002** |

***Men vs. women by chi-square test.**

**Suppl Table 2. Distribution of participants' responses to environmentally responsible food choices items according to sex and Mediterranean Diet adherence**

| **Environmentally friendly food preference scale items** | **Total** | | | | | | **Men** | | | | | | **Women** | | | | | | **p** |
| --- | --- | --- | --- | --- | --- | --- | --- | --- | --- | --- | --- | --- | --- | --- | --- | --- | --- | --- | --- |
|  | **Low** | | **Moderate** | | **High** | | **Low** | | **Moderate** | | **High** | | **Low** | | **Moderate** | | **High** | |  |
|  | **n** | **%** | **n** | **%** | **n** | **%** | **n** | **%** | **n** | **%** | **n** | **%** | **n** | **%** | **n** | **%** | **n** | **%** |  |
| **I can pay more for organically grown food** | | | | | | | | | | | | | | | | | | |  |
| **Never** | 32^a^ | 7,5 | 39^b^ | 4,3 | 10^b^ | 2,5 | 14^a^ | 8,2 | 19^a^ | 6,1 | 8^a^ | 6,1 | 18^a^ | 7,0 | 20^b^ | 3,4 | 2^c^ | 0,7 |  |
| **Rarely** | 59^a^ | 13,8 | 117^a^ | 12,9 | 45^a^ | 11,3 | 18^a^ | 10,6 | 44^a^ | 14,0 | 14^a^ | 10,7 | 41^a^ | 16,0 | 73^a^ | 12,3 | 31^a^ | 11,6 |  |
| **Sometimes** | 137^a^ | 32,1 | 288^a^ | 31,8 | 131^a^ | 32,8 | 58^a^ | 34,1 | 81^a^ | 25,8 | 41^a^ | 31,3 | 79^a^ | 30,7 | 207^a^ | 35,0 | 90^a^ | 33,6 |  |
| **Generally** | 136^a^ | 31,9 | 291^a^ | 32,1 | 146^a^ | 36,6 | 53^a^ | 31,2 | 112^a^ | 35,7 | 44^a^ | 33,6 | 83^a^,^b^ | 32,3 | 179^b^ | 30,2 | 102^a^ | 38,1 |  |
| **Always** | 63^a^ | 14,8 | 171^a^ | 18,9 | 67^a^ | 16,8 | 23^a^ | 15,9 | 58^a^ | 18,5 | 24^a^ | 18,3 | 36^a^ | 14,0 | 113^a^ | 19,1 | 43^a^ | 16,0 |  |
|  | **p=0,025** | | | | | | **p=0,620** | | | | | | **p=0,002** | | | | | | **p=0,025** |
| **I avoid consuming food with GMO (genetically modified organism)** | | | | | | | | | | | | | | | | | | |  |
| **Never** | 24^a^ | 5,6 | 35^a^ | 3,9 | 7^b^ | 1,8 | 13^a^ | 7,6 | 17^a^,^b^ | 5,4 | 3^b^ | 2,3 | 11^a^ | 4,3 | 18^a^ | 3,0 | 4^a^ | 1,5 |  |
| **Rarely** | 68^a^ | 15,9 | 85^b^ | 9,4 | 47^a^,^b^ | 11,8 | 34^a^ | 20,0 | 27^b^ | 8,6 | 21^a^ | 16,0 | 34^a^ | 13,2 | 58^a^ | 9,8 | 26^a^ | 9,7 |  |
| **Sometimes** | 106^a^ | 24,8 | 171^b^ | 18,9 | 59^b^ | 14,8 | 33^a^ | 19,4 | 57^a^ | 18,2 | 20^a^ | 15,3 | 73^a^ | 28,4 | 114^b^ | 19,3 | 39^b^ | 14,6 |  |
| **Generally** | 146^a^ | 34,2 | 376^b^ | 41,5 | 154^a^,^b^ | 38,6 | 56^a^ | 32,9 | 121^a^ | 38,5 | 40^a^ | 30,5 | 90^a^ | 35,0 | 255^b^ | 43,1 | 114^a^,^b^ | 42,5 |  |
| **Always** | 83^a^ | 19,4 | 239^b^ | 26,4 | 132^c^ | 33,1 | 34^a^ | 20,0 | 92^b^ | 29,3 | 47^b^ | 25,9 | 49^a^ | 19,1 | 147^a^ | 24,8 | 85^b^ | 31,7 |  |
|  | **p=0,000** | | | | | | **p=0,001** | | | | | | **p=0,000** | | | | | | **p=0,000** |
| **I prefer to consume eco-label food** | | | | | | | | | | | | | | | | | | |  |
| **Never** | 39^a^ | 9,1 | 59^a^ | 6,5 | 25^a^ | 6,3 | 15^a^ | 8,8 | 21^a^ | 6,7 | 16^a^ | 12,2 | 24^a^ | 9,3 | 38^a^,^b^ | 6,4 | 9^b^ | 3,4 |  |
| **Rarely** | 86^a^ | 20,1 | 125^b^ | 13,8 | 59^b^ | 14,8 | 34^a^ | 20,0 | 39^b^ | 12,4 | 18^a^,^b^ | 13,7 | 52^a^ | 20,2 | 86^b^ | 14,5 | 41^a^,^b^ | 15,3 |  |
| **Sometimes** | 148^a^ | 34,7 | 285^a^ | 31,5 | 101^b^ | 25,3 | 58^a^ | 34,1 | 91^a^ | 29,0 | 38^a^ | 29,0 | 90^a^ | 35,0 | 194^a^ | 32,8 | 63^b^ | 23,5 |  |
| **Generally** | 124^a^ | 29,0 | 313^b^ | 34,5 | 138^a^,^b^ | 34,6 | 51^a^ | 30,0 | 127^b^ | 40,4 | 32^a^ | 24,4 | 73^a^ | 28,4 | 186^a^ | 31,4 | 106^b^ | 39,6 |  |
| **Always** | 30^a^ | 7,0 | 124^b^ | 13,7 | 76^c^ | 19,0 | 12^a^ | 7,1 | 36^a^ | 11,5 | 27^b^ | 20,6 | 18^a^ | 7,0 | 88^b^ | 14,9 | 49^b^ | 18,3 |  |
|  | **p=0,000** | | | | | | **p=0,000** | | | | | | **p=0,000** | | | | | | **p=0,000** |
| **I am careful not to consume too much meat** | | | | | | | | | | | | | | | | | | |  |
| **Never** | 106^a^ | 24,8 | 157^b^ | 17,3 | 47^c^ | 11,8 | 46^a^ | 27,1 | 67^a^ | 21,3 | 24^a^ | 18,3 | 60^a^ | 23,3 | 90^b^ | 15,2 | 23c | 8,6 |  |
| **Rarely** | 123^a^ | 28,8 | 223^a^ | 24,6 | 98^a^ | 24,6 | 46^a^ | 27,1 | 76^a^ | 24,2 | 34^a^ | 26,0 | 77^a^ | 30,0 | 147^a^ | 24,8 | 64^a^ | 23,9 |  |
| **Sometimes** | 109^a^ | 25,5 | 292^b^ | 32,2 | 138^b^ | 34,6 | 45^a^ | 26,5 | 99^a^ | 31,5 | 38^a^ | 29,0 | 64^a^ | 24,9 | 193^b^ | 32,6 | 100^b^ | 37,3 |  |
| **Generally** | 74^a^ | 17,3 | 189^a^,^b^ | 20,9 | 91^b^ | 22,8 | 25^a^ | 14,7 | 56^a^ | 17,8 | 27^a^ | 20,6 | 49^a^ | 19,1 | 133^a^ | 22,5 | 64^a^ | 23,9 |  |
| **Always** | 15^a^ | 3,5 | 45^a^ | 5,0 | 25^a^ | 6,3 | 8^a^ | 4,7 | 16^a^ | 5,1 | 8^a^ | 6,1 | 7^a^ | 2,7 | 29^a^,^b^ | 4,9 | 17^b^ | 6,3 |  |
|  | **p=0,000** | | | | | | **p=0,655** | | | | | | **p=0,000** | | | | | | **p=0,000** |
| **I prefer to buy dairy products from local producers** | | | | | | | | | | | | | | | | | | |  |
| **Never** | 43^a^ | 10,1 | 64^a^,^b^ | 7,1 | 19^b^ | 4,8 | 17^a^ | 10,0 | 25^a^ | 8,0 | 8^a^ | 6,1 | 26^a^ | 10,1 | 39^a^,^b^ | 6,6 | 11^b^ | 4,1 |  |
| **Rarely** | 72^a^ | 16,9 | 143^a^ | 15,8 | 65^a^ | 19,3 | 22^a^ | 12,9 | 42^a^ | 13,4 | 18^a^ | 13.7 | 50^a^ | 19,5 | 101^a^ | 17,1 | 47^a^ | 17,5 |  |
| **Sometimes** | 139^a^ | 32,6 | 234^b^ | 25,8 | 89^b^ | 22,3 | 60^a^ | 35,3 | 83^b^ | 26,4 | 29^b^ | 22,1 | 79^a^ | 30,7 | 151^a^,^b^ | 25,5 | 60^b^ | 22,4 |  |
| **Generally** | 112^a^ | 26,2 | 320^b^ | 35,3 | 143^b^ | 35,8 | 45^a^ | 26,5 | 127^b^ | 40,4 | 51^b^ | 38,9 | 67^a^ | 26,1 | 193^a^,^b^ | 32,6 | 92^b^ | 34,3 |  |
| **Always** | 61^a^ | 14,3 | 145^a^ | 16,0 | 83^b^ | 20,8 | 26^a^,^b^ | 15,3 | 37^b^ | 11,8 | 25^a^ | 19,1 | 35^a^ | 13,6 | 108^a^,^b^ | 18,2 | 58^b^ | 21,6 |  |
|  | **p=0,000** | | | | | | **p=0,037** | | | | | | **p=0,013** | | | | | | **p=0,000** |
| **I avoid consuming export food such as a variety of exotic fruits** | | | | | | | | | | | | | | | | | | |  |
| **Never** | 74^a^ | 17,3 | 171^a^ | 18,9 | 49 | 12,3 | 29^a^ | 17,1 | 59^a^ | 18,8 | 18^a^ | 13,7 | 45^a^,^b^ | 17,5 | 112^b^ | 18,9 | 31^a^ | 11,6 |  |
| **Rarely** | 110^a^ | 25,8 | 199^a^ | 22,0 | 107^a^ | 26,8 | 44^a^ | 25,9 | 53^b^ | 16,9 | 32^a^,^b^ | 24,4 | 66^a^ | 25,7 | 146^a^ | 24,7 | 75^a^ | 28,0 |  |
| **Sometimes** | 117^a^ | 27,4 | 251^a^ | 27,7 | 116^a^ | 29,1 | 47^a^ | 27,6 | 96^a^ | 30,6 | 36^a^ | 27,5 | 70^a^ | 27,2 | 155^a^ | 26,2 | 80^a^ | 29,9 |  |
| **Generally** | 103^a^ | 24,1 | 208^a^ | 23,0 | 88^a^ | 22,1 | 42^a^ | 24,7 | 74^a^ | 23,6 | 30^a^ | 22,9 | 61^a^ | 23,7 | 134^a^ | 22,6 | 58^a^ | 21,6 |  |
| **Always** | 23^a^ | 5,4 | 77^b^ | 8,5 | 39^b^ | 9,8 | 8^a^ | 4,7 | 32^b^ | 10,2 | 15^b^ | 11,5 | 15^a^ | 5,8 | 45^a^ | 7,6 | 24^a^ | 9,0 |  |
|  | **p=0,034** | | | | | | **p=0,149** | | | | | | **p=0,288** | | | | | | **p=0,034** |
| **I avoid consuming canned “ready-made” food** | | | | | | | | | | | | | | | | | | |  |
| **Never** | 53^a^ | 2,4 | 76^b^ | 8,4 | 15^c^ | 3,8 | 18^a^ | 10,6 | 29^a^ | 9,2 | 5^b^ | 3,8 | 35^a^ | 13,6 | 47^b^ | 7,9 | 10^c^ | 3,7 |  |
| **Rarely** | 98^a^ | 23,0 | 179^a^,^b^ | 19,8 | 67^b^ | 16,8 | 46^a^ | 27,1 | 67^a^ | 21,3 | 23^a^ | 17,6 | 52^a^ | 20,2 | 112^a^ | 18,9 | 44^a^ | 16,4 |  |
| **Sometimes** | 134^a^ | 31,4 | 22^b^ | 24,5 | 92^b^ | 23,1 | 43^a^ | 25,3 | 73^a^ | 23,2 | 32^a^ | 24,4 | 91^a^ | 35,4 | 149^b^ | 25,2 | 60^b^ | 22,4 |  |
| **Generally** | 100^a^ | 23,4 | 313^b^ | 34,5 | 162^c^ | 40,6 | 49^a^ | 28,8 | 107^a^ | 34,1 | 49^a^ | 37,4 | 51^a^ | 19,8 | 206^b^ | 34,8 | 113^c^ | 42,2 |  |
| **Always** | 42^a^ | 9,8 | 116^a^,^b^ | 12,8 | 63^b^ | 15,8 | 14^a^ | 8,2 | 38^a^,^b^ | 12,1 | 22^b^ | 16,8 | 28^a^ | 10,9 | 78^a^ | 13,2 | 41^a^ | 15,3 |  |
|  | **p=0,000** | | | | | | **p=0,079** | | | | | | **p=0,000** | | | | | | **p=0,000** |

***Men vs. women by chi-square test.**

**Suppl Table 3. Stratification of adherence to MEDAS items by sex.**

| **MEDAS items** | **Total** | | | | | | **Men** | | | | | | **Women** | | | | | | **p** |
| --- | --- | --- | --- | --- | --- | --- | --- | --- | --- | --- | --- | --- | --- | --- | --- | --- | --- | --- | --- |
|  | **Low** | | **Moderate** | | **High** | | **Low** | | **Moderate** | | **High** | | **Low** | | **Moderate** | | **High** | |  |
|  | **n** | **%** | **n** | **%** | **n** | **%** | **n** | **%** | **n** | **%** | **n** | **%** | **n** | **%** | **n** | **%** | **n** | **%** |  |
| **Using olive oil as main culinary fat** | | | | | | | | | | | | | | | | | | | |
| **Yes** | 223^a^ | 52,2 | 771^b^ | 85,1 | 386^c^ | 96,7 | 78^a^ | 45,9 | 268^b^ | 85,4 | 124^c^ | 94,7 | 145^a^ | 56,4 | 503^b^ | 85,0 | 262^b^ | 87,8 |  |
| **No** | 204^a^ | 47,8 | 135^b^ | 14,9 | 13^c^ | 3,3 | 92^a^ | 54,1 | 46^b^ | 14,6 | 7^c^ | 5,3 | 112^a^ | 43,6 | 89^b^ | 15,0 | 6^c^ | 2,2 |  |
|  | **p=0,000** | | | | | | **p=0,000** | | | | | | **p=0,000** | | | | | | **p=0,013** |
| **Quantity of olive oil per day** | | | | | | | | | | | | | | | | | | | |
| **Yes** | 81^a^ | 19,0 | 422^b^ | 46,6 | 289^c^ | 72,4 | 21^a^ | 12,4 | 132^b^ | 42,0 | 94^c^ | 71,8 | 60^a^ | 23,3 | 290^b^ | 49,0 | 195^c^ | 72,8 |  |
| **No** | 346^a^ | 81,0 | 484^b^ | 53,4 | 110^c^ | 27,6 | 149^a^ | 87,6 | 182^b^ | 58,0 | 37^c^ | 28,2 | 197^a^ | 76,7 | 302^b^ | 51,0 | 73^c^ | 27,2 |  |
|  | **p=0,000** | | | | | | **p=0,000** | | | | | | **p=0,000** | | | | | | **p=0,001** |
| **Servings of vegetables per day** | | | | | | | | | | | | | | | | | | | |
| **Yes** | 38^a^ | 8,9 | 276^b^ | 30,5 | 251^c^ | 62,9 | 10^a^ | 5,91 | 68^b^ | 21,7 | 70^c^ | 53,4 | 28^a^ | 10,9 | 208^b^ | 35,1 | 181^c^ | 67,5 |  |
| **No** | 389^a^ | 91,1 | 630^b^ | 69,5 | 148^c^ | 37,1 | 60^a^ | 94,1 | 246^b^ | 78,3 | 61^c^ | 46,6 | 229^a^ | 89,1 | 384^b^ | 64,9 | 87^c^ | 32,5 |  |
|  | **p=0,000** | | | | | | **p=0,000** | | | | | | **p=0,000** | | | | | | **p=0,000** |
| **Servings of fruits per day** | | | | | | | | | | | | | | | | | | | |
| **Yes** | 45^a^ | 10,5 | 208^b^ | 23,0 | 173^c^ | 43,4 | 21^a^ | 12,4 | 83^b^ | 26,4 | 60^c^ | 45,8 | 24^a^ | 9,3 | 125^b^ | 21,1 | 113^c^ | 42,2 |  |
| **No** | 382^a^ | 89,5 | 698^b^ | 77,0 | 226^c^ | 56,6 | 149^a^ | 87,6 | 231^b^ | 73,6 | 71^c^ | 54,2 | 233^a^ | 90,7 | 467^b^ | 78,9 | 155^c^ | 57,8 |  |
|  | **p=0,000** | | | | | | **p=0,000** | | | | | | **p=0,000** | | | | | | p=0,138 |
| **Servings of red meat per day** | | | | | | | | | | | | | | | | | | | |
| **Yes** | 229^a^ | 53,6 | 617^b^ | 68,1 | 319^c^ | 79,9 | 88^a^ | 51,8 | 214^b^ | 68,2 | 94^b^ | 71,8 | 141^a^ | 54,9 | 403^b^ | 68,1 | 225^c^ | 84,0 |  |
| **No** | 198^a^ | 46,4 | 289^b^ | 31,9 | 80^c^ | 20,1 | 82^a^ | 48,2 | 100^b^ | 31,8 | 37^b^ | 28,2 | 116^a^ | 45,1 | 189^b^ | 31,9 | 43^c^ | 16,0 |  |
|  | **p=0,000** | | | | | | **p=0,000** | | | | | | **p=0,000** | | | | | | p=0,059 |
| **Servings of butter or margarine per day** | | | | | | | | | | | | | | | | | | | |
| **Yes** | 210^a^ | 49,2 | 645^b^ | 71,2 | 351^c^ | 88,0 | 91^a^ | 53,5 | 22^b^ | 72,0 | 118^c^ | 90,1 | 119^a^ | 46,3 | 419^b^ | 70,8 | 233^c^ | 86,9 |  |
| **No** | 217^a^ | 50,8 | 261^b^ | 28,8 | 48^c^ | 12,0 | 79^a^ | 46,5 | 88^b^ | 28,0 | 13^c^ | 9,9 | 138^a^ | 53,7 | 173^b^ | 29,2 | 35^c^ | 13,1 |  |
|  | **p=0,000** | | | | | | **p=0,000** | | | | | | **p=0,000** | | | | | | p=0,460 |
| **Servings of sweet or carbonated beverages per day** | | | | | | | | | | | | | | | | | | | |
| **Yes** | 197^a^ | 46,1 | 673^b^ | 74,3 | 364^c^ | 91,2 | 71^a^ | 41,8 | 214^b^ | 68,2 | 118^c^ | 90,1 | 126^a^ | 49,0 | 459^b^ | 77,5 | 246^c^ | 91,8 |  |
| **No** | 230^a^ | 53,9 | 233^b^ | 25,7 | 35^c^ | 8,8 | 99^a^ | 58,2 | 100^b^ | 31,8 | 13^c^ | 9,9 | 131^a^ | 51,0 | 133^b^ | 22,5 | 22^c^ | 8,2 |  |
|  | **p=0,000** | | | | | | **p=0,000** | | | | | | **p=0,000** | | | | | | **p=0,000** |
| **Wine portion per week** | | | | | | | | | | | | | | | | | | | |
| **Yes** | 12^a^ | 2,8 | 48^b^ | 5,3 | 56^c^ | 14,0 | 8^a^ | 4,7 | 33^b^ | 10,5 | 30^c^ | 22,9 | 4^a^ | 1,6 | 15^a^ | 2,5 | 26^b^ | 9,7 |  |
| **No** | 415^a^ | 97,2 | 858^b^ | 94,7 | 343^c^ | 86,0 | 162^a^ | 95,3 | 281^b^ | 89,5 | 101^c^ | 77,1 | 253^a^ | 98,4 | 577^a^ | 97,5 | 242^b^ | 90,3 |  |
|  | **p=0,000** | | | | | | **p=0,000** | | | | | | **p=0,000** | | | | | | **p=0,000** |
| **Servings of legumes per week** | | | | | | | | | | | | | | | | | | | |
| **Yes** | 113^a^ | 26,5 | 407^b^ | 44,9 | 292^c^ | 73,2 | 57^a^ | 33,5 | 159^b^ | 50,6 | 109^c^ | 83,2 | 56^a^ | 21,8 | 248^b^ | 41,9 | 183^c^ | 68,3 |  |
| **No** | 314^a^ | 73,5 | 499^b^ | 55,1 | 107^c^ | 26,8 | 113^a^ | 66,5 | 155^b^ | 49,4 | 22^c^ | 16,8 | 201^a^ | 78,2 | 344^b^ | 58,1 | 85^c^ | 31,7 |  |
|  | **p=0,000** | | | | | | **p=0,000** | | | | | | **p=0,000** | | | | | | **p=0,000** |
| **Servings of fresh, frozen or canned fish per week** | | | | | | | | | | | | | | | | | | | |
| **Yes** | 24^a^ | 5,6 | 132^b^ | 14,6 | 149^c^ | 37,3 | 13^a^ | 7,6 | 65^b^ | 20,7 | 55^c^ | 42,0 | 11^a^ | 4,3 | 67^b^ | 11,3 | 94^c^ | 35,1 |  |
| **No** | 403^a^ | 94,4 | 774^b^ | 85,4 | 250^c^ | 62,7 | 157^a^ | 92,4 | 249^b^ | 79,3 | 76^c^ | 58,0 | 246^a^ | 95,7 | 525^b^ | 88,7 | 174^c^ | 64,9 |  |
|  | **p=0,000** | | | | | | **p=0,000** | | | | | | **p=0,000** | | | | | | **p=0,001** |
| **Servings of commercial sweets per week** | | | | | | | | | | | | | | | | | | | |
| **Yes** | 215^a^ | 50,4 | 718^b^ | 79,2 | 357^c^ | 89,5 | 91^a^ | 53,5 | 242^b^ | 77,1 | 115^c^ | 87,8 | 124^a^ | 48,2 | 476^b^ | 80,4 | 242^c^ | 90,3 |  |
| **No** | 212^a^ | 49,6 | 188^b^ | 20,8 | 42^c^ | 10,5 | 79^a^ | 46,5 | 72^b^ | 22,9 | 16^c^ | 12,2 | 133^a^ | 51,8 | 116^b^ | 19,6 | 26^c^ | 9,7 |  |
|  | **p=0,000** | | | | | | **p=0,000** | | | | | | **p=0,000** | | | | | | p=0,247 |
| **Servings of nuts per week** | | | | | | | | | | | | | | | | | | | |
| **Yes** | 111^a^ | 26,0 | 373^b^ | 41,2 | 288^c^ | 72,2 | 54^a^ | 31,8 | 148^b^ | 47,1 | 97^c^ | 74,0 | 57^a^ | 22,2 | 225^b^ | 38,0 | 191^c^ | 71,3 |  |
| **No** | 316^a^ | 74,0 | 533^b^ | 58,8 | 11^c^ | 27,8 | 116^a^ | 68,2 | 166^b^ | 52,9 | 34^c^ | 26,0 | 200^a^ | 77,8 | 367^b^ | 62,0 | 77^c^ | 28,7 |  |
|  | **p=0,000** | | | | | | **p=0,000** | | | | | | **p=0,000** | | | | | | **p=0,012** |
| **Consumption preferences of chicken, turkey, or rabbit meat instead of hamburger and sausage** | | | | | | | | | | | | | | | | | | | |
| **Yes** | 128^a^ | 30,0 | 429^b^ | 47,4 | 271^c^ | 67,9 | 48^a^ | 28,2 | 143^b^ | 45,5 | 85^c^ | 64,9 | 80^a^ | 31,1 | 286^b^ | 48,3 | 186^c^ | 69,4 |  |
| **No** | 299^a^ | 70,0 | 477^b^ | 52,6 | 128^c^ | 32,1 | 122^a^ | 71,8 | 171^b^ | 54,5 | 46^c^ | 35,1 | 177^a^ | 68,9 | 306^b^ | 51,7 | 82^c^ | 30,6 |  |
|  | **p=0,000** | | | | | | **p=0,000** | | | | | | **p=0,000** | | | | | | p=0,070 |
| **Servings of vegetables, pasta, rice with olive oil, onion, garlic and tomato per week** | | | | | | | | | | | | | | | | | | | |
| **Yes** | 183^a^ | 42,9 | 601^b^ | 66,3 | 317^c^ | 79,4 | 59^a^ | 34,7 | 195^b^ | 62,1 | 89^b^ | 67,9 | 124^a^ | 48,2 | 406^b^ | 68,6 | 228^c^ | 85,1 |  |
| **No** | 244^a^ | 57,1 | 305^b^ | 33,7 | 82^c^ | 20,6 | 111^a^ | 65,3 | 119^b^ | 37,9 | 42^b^ | 32,1 | 133^a^ | 51,8 | 186^b^ | 31,4 | 40^c^ | 14,9 |  |
|  | **p=0,000** | | | | | | **p=0,000** | | | | | | **p=0,000** | | | | | | **p=0,000** |

***Men vs. women by chi-square test.**

**Suppl Table 4. Correlation of some parameters with sustainable nutrition knowledge score and environmentally sensitive food preference scores according to the Mediterranean Diet adherence score of the participants**

|  | **Mediterranean Diet Adherence Score** | |
| --- | --- | --- |
|  | **r** | **p*** |
| **Age** | 0,093 | **0,000**** |
| **BMI** | 0,04 | 0,872 |
| **Education time** | 0,022 | 0,370 |
| **Sustainable nutrition knowledge score** | 0,140 | **0,000**** |
| **Environmentally responsible food preference score** | 0,180 | **0,000**** |

* Pearson correlation test **P < 0.001
